# G-CSF Prevents Progression of Diabetic Nephropathy in Rat

**DOI:** 10.1371/journal.pone.0077048

**Published:** 2013-10-22

**Authors:** Byung-Im So, Yi-Sun Song, Cheng-Hu Fang, Jun-Young Park, Yonggu Lee, Jeong Hun Shin, Hyuck Kim, Kyung-Soo Kim

**Affiliations:** 1 Graduate School of Biomedical Science and Engineering, Hanyang University, Seoul, Korea; 2 Department of Internal Medicine, Hanyang University College of Medicine, Seoul, Korea; 3 Department of Internal Medicine, Yanbian University, College of Medicine, Yanji, China; 4 Department of Thoracic and Cardiovascular Surgery, Hanyang University College of Medicine, Seoul, Korea; Institut National de la Santé et de la Recherche Médicale, France

## Abstract

**Background:**

The protective effects of granulocyte colony-stimulating factor (G-CSF) have been demonstrated in a variety of renal disease models. However, the influence of G-CSF on diabetic nephropathy (DN) remains to be examined. In this study, we investigated the effect of G-CSF on DN and its possible mechanisms in a rat model.

**Methods:**

Otsuka Long-Evans Tokushima Fatty (OLETF) rats with early DN were administered G-CSF or saline intraperitoneally. Urine albumin creatinine ratio (UACR), creatinine clearance, mesangial matrix expansion, glomerular basement membrane (GBM) thickness, and podocyte foot process width (FPW) were measured. The levels of interleukin (IL)-1β, transforming growth factor (TGF)-β1, and type IV collagen genes expression in kidney tissue were also evaluated. To elucidate the mechanisms underlying G-CSF effects, we also assessed the expression of G-CSF receptor (G-CSFR) in glomeruli as well as mobilization of bone marrow (BM) cells to glomeruli using sex-mismatched BM transplantation.

**Results:**

After four weeks of treatment, UACR was lower in the G-CSF treatment group than in the saline group (*p*<0.05), as were mesangial matrix expansion, GBM thickness, and FPW (*p*<0.05). In addition, the expression of TGF-β1 and type IV collagen and IL-1β levels was lower in the G-CSF treatment group (*p*<0.05). G-CSFR was not present in glomerular cells, and G-CSF treatment increased the number of BM-derived cells in glomeruli (*p*<0.05).

**Conclusions:**

G-CSF can prevent the progression of DN in OLETF rats and its effects may be due to mobilization of BM cells rather than being a direct effect.

## Introduction

Diabetes mellitus (DM) is a multisystem disorder that affects various organs. Almost 30% of DM patients develop diabetic nephropathy (DN), despite control of blood glucose and/or blood pressure. DN is widely recognized as a common cause of end-stage renal disease [1], [2]. It is characterized clinically by proteinuria accompanied by decreased glomerular filtration rate (GFR) [1], [3]. In addition, it is histologically defined by glomerular basement membrane (GBM) thickening and mesangial matrix expansion with the accumulation of extracellular matrix proteins [4], [5].

Granulocyte colony-stimulating factor (G-CSF) is frequently used to mobilize hematopoietic stem cells from the bone marrow (BM) into the peripheral blood [6]. In a previous clinical study, G-CSF was employed to accelerate recovery from febrile neutropenia after cytotoxic therapy and to harvest donor cells in peripheral blood after BM transplantation (BMT) [7]. Recently, experimental studies have demonstrated the beneficial effects of G-CSF in the kidney, brain, liver, and heart. For example, one study showed that G-CSF increased angiogenesis and reversed ischemic damage in the brain [8]. In other studies, G-CSF protected against renal tubular injury induced by adriamycin [9] and against hepatic steatosis [10], as well as diabetic cardiomyopathy in rats [11]. Flaquer M, et al. demonstrated that the combination of hepatocyte growth factor gene therapy with hematopoietic stem cell mobilization by G-CSF may contribute to renal tissue repair and regeneration in diabetes- induced mice [12]. However, the effect of G-CSF itself on DN is unknown. In this study, we evaluated the effects of G-CSF on early DN using Otsuka Long-Evans Tokushima Fatty (OLETF) rats and investigated possible mechanisms underlying the beneficial effects.

## Materials and Methods

### Animals

The experiments were performed in compliance with the ARRIVE guidelines on animal research [13], and all protocols were approved by the Hanyang University Institutional Animal Care and Use Committee. We used OLETF rats and control Long-Evans Tokushima Otsuka (LETO) rats, supplied by Otsuka Pharmaceutical Co. (Tokushima, Japan). OLETF rats were developed as spontaneous long-term hyperglycemic rats with type 2 DM; they have also been used to model aspects of metabolic syndrome, such as obesity and fatty liver [14]. The rats were kept in a specific pathogen-free facility at the Hanyang University Medical School Animal Experiment Center at a controlled temperature (23°C±2°C) and humidity (55%±5%), with a 12-hr artificial light/dark cycle.

### Development of the DN Model

**Figure 1:**
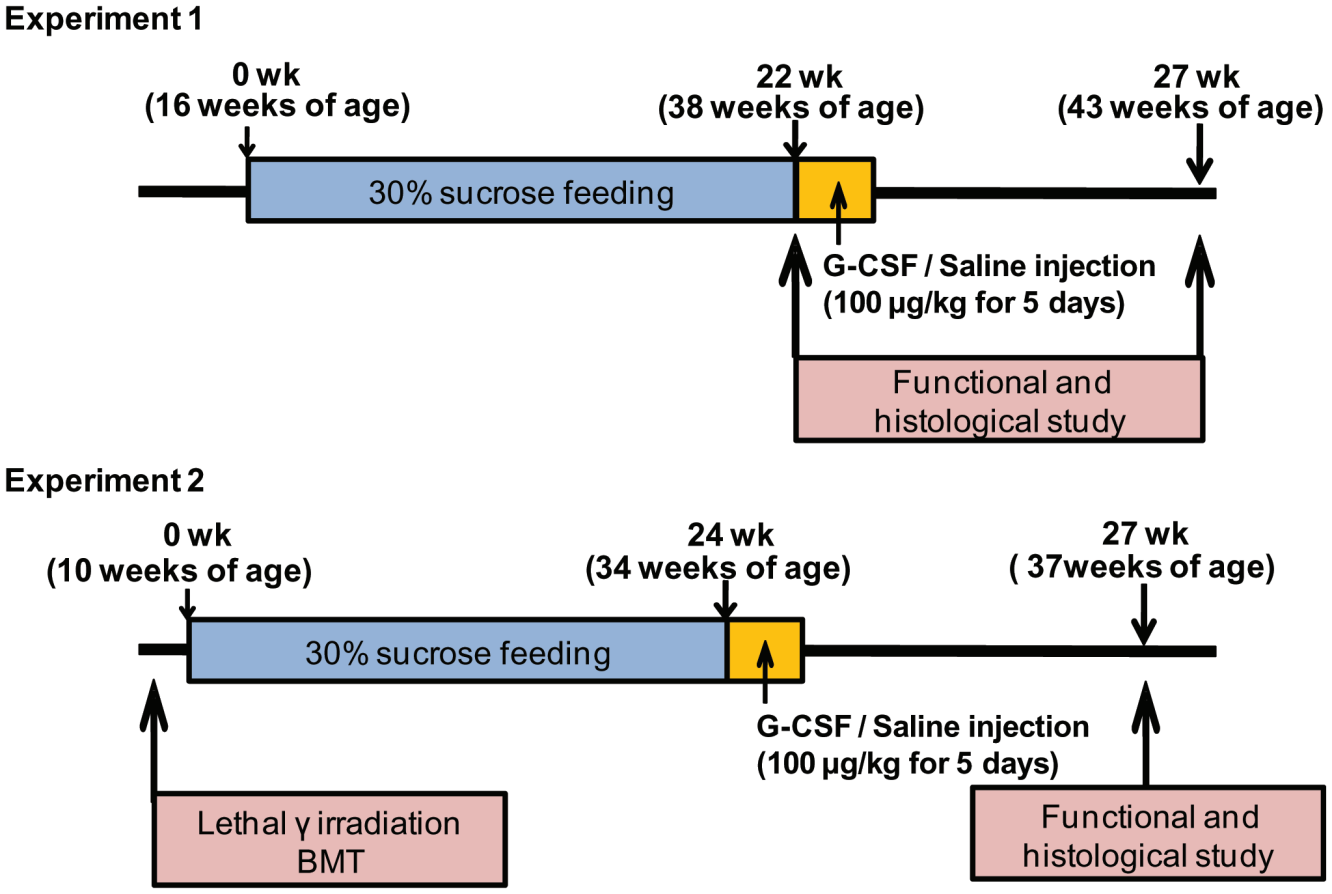
Experimental design. Experiment 1: a rat model of diabetic nephropathy (male OLETF rats), Experiment 2: a rat model of diabetic nephropathy with bone marrow transplantation (BMT) (donors: male OLETF rats, recipients: female OLETF rats).

Starting at 16 weeks of age, all male OLETF rats (*n* = 12) received water containing 30% sucrose *ad libitum* to facilitate the development of DN. After DN had been induced, they received no more sucrose water. LETO rats (*n* = 7), as normal controls, received tap water without 30% sucrose. The DN model was confirmed by serum fasting glucose level exceeding 200 mg/dl, urine albumin creatinine ratio (UACR) exceeding 300 mg/g, and histologically-confirmed GBM thickening and mesangial matrix expansion [15]–[17]. To detect the development of the DN model, body weights, serum glucose levels, and urine albumin levels of all the rats were measured at 4 week intervals from 16 to 38 weeks of age. After 22 weeks of sucrose feeding (at 38 weeks of age), three male OLETF and three male LETO rats were sacrificed for histological examination. One OLETF rat died during development of the DN model. To test the effect of G-CSF on DN, we used eight male OLETF rats and four male LETO rats. The experimental design is outlined in Fig. 1.

### Bone Marrow Transplantation Model

Starting at 6 weeks of age, female OLETF rats received lethal γ-irradiation (3 and 4 Gy, 4 hr apart, total of 7 Gy) from a Gammacell 1000 Elite (MDS Nordion, Ottawa, Canada) [18], [19]. On the following day, 2×10^8^ donor BM cells from male OLETF rats were administered intravenously via the tail vein. Donor BM cells were obtained as described previously [20]. To confirm the DN model, female OLETF rats (after 4 weeks of recovery from BMT; 10 weeks of age) received tap water containing 30% sucrose *ad libitum* and certified rodent laboratory chow. After 24 weeks of sucrose feeding (at 34 weeks of age), DN was confirmed in the female rats (data not shown). The experimental design is outlined in Fig. 1.

### G-CSF Administration

OLETF rats in which DN had been induced were divided randomly into two groups (G-CSF-treated and saline-treated, *n* = 4 each), treated daily for 5 days with G-CSF (100 µg/kg per day, Dong-A Pharmacological, Seoul, Korea) or an equal volume of saline (Dai Han Pharm. Co., Ltd., Seoul, Korea), and observed for 4 weeks [21].

### Urine and Blood Chemistry

Before and after G-CSF treatment, we measured serum glucose, total cholesterol (TC), triglyceride (TG), UACR, and creatinine clearance (CrCl). Animals were transferred to individual metabolic cages for urine sample collection, and 24-hours urine samples were collected to measure UACR levels [22]. Blood samples were collected from the tail vein after 8 hours of fasting, and serum glucose, TC, and TG levels were analyzed with an Olympus AU400 auto analyzer (Olympus GmbH, Hamburg, Germany) [23].

### Histological Examination

Kidney tissue from each animal was fixed in 10% formalin solution (pH 7.4) and embedded in paraffin. The embedded tissue was cut into 3- µm-thick sections and subjected to periodic acid-Schiff (PAS) staining for light microscopic evaluation. Mesangial matrix expansion in the glomeruli was evaluated in PAS-stained sections using Image-Pro Plus 4.5 (Media Cybernetics, Silver Spring, MD) [24]. The mean percent area of PAS-stained glomeruli was calculated for 20 randomly selected fields of each kidney section.

### Ultrastructural Examination

For electron microscope evaluation, the kidneys were fixed in 2.5% glutaraldehyde (0.2 M cacodylate buffer, pH 7.4) and embedded in epoxy resin. Ultrathin sections were double-stained with 1.25% uranium acetate and 0.4% lead citrate and then observed with an electron microscope (H-7600 s, Nikon, Tokyo, Japan). GBM thickness was determined in the glomeruli where the epithelial and endothelial cells were clearly visible. A perpendicular line of GBM was drawn from the endothelial to the epithelial edge, and it was measured by image analysis using Image-Pro Plus 4.5 (Media Cybernetics, Silver Spring, MD) [25]. The mean GBM thickness was calculated for 20 randomly selected fields of each kidney section. To examine podocyte foot process width (FPW), the mean of FPW was calculated as described previously [26], [27]: FPW = π/4×∑ GBM length/∑ foot process. ∑ GBM length is the total peripheral GBM length in each image, and ∑ foot process is the total foot processes number on peripheral GBM in each image. The correction factor π/4 was used to correct for presumed random variation in the angle of section relative to the long axis of the podocyte. The mean of FPW was determined from the images at 10,000× magnification of each kidney section.

### Reverse Transcriptase-polymerase Chain Reaction (RT-PCR) Analysis of G-CSF Receptor (G-CSFR) Gene Expression

Kidneys were frozen in liquid nitrogen and stored at −80°C for the RT-PCR assays. Total RNA was extracted using TRIzol (Invitrogen, Carlsbad, USA), and then reverse transcribed using SuperScript II reverse transcriptase (Invitrogen, Carlsbad, USA) according to the manufacturer’s protocol. We performed RT-PCR to analyze G-CSFR and β-actin. The sequences of the rat primer pairs for G-CSFR and β-actin and the original clones are listed in Table 1. PCR was carried out for 30 cycles of denaturation at 90°C for 30 sec, annealing at 60°C for 30 sec, and extension at 72°C for 1 min. The RT-PCR products were visualized by electrophoresis on 1.5% (w/v) agarose gels using Gel-Doc 2000 (Bio-Rad, CA, USA).

**Table 1:** Sequences of primers.

Primer	Sequences	Size(bp)
G-CSFR	F: 5′-CCA-TTG-TCC-ATC-TTG-GGG-ATC-3′	234
	R: 5′-CCT-GGA-AGC-TGT-TGT-TCC-ATG-3′	
β-actin	F: 5′-ACC-TTC-AAC-AAC-CCA-GCC-ATG-TAC-G-3′	698
	R: 5′-CTG-ATC-CAC-ATC-TGC-TGG-AAG-GTG-G-3′	
TGF-β1	F: 5′-TGT-TCT-TCA-ATA-CGT-CAG-ACA-TTC-G-3′	102
	R: 5′-GTT-GCT-CCA-CAG-TTG-ACT-TGA-ATC-T-3′	
Type IVcollagen	F: 5′-GAG-GGT-GCT-GGA-CAA-GCT-CTT-3′	67
	R: 5′-TAA-ATG-GAC-TGG-CTC-GGA-ATT-C-3′	
IL-1β	F: 5′-AAT-GAC-CTG-TTC-TTT-GAG-GCT-GAC-3′	115
	R: 5′-CGA-GAT-GCT-GCT-GTG-AGA-TTT-GAA-G-3′	
GAPDH	F: 5′-CCT-TCT-CTT-GTG-ACA-AAG-TGG-ACA-T-3′	96
	R: 5′-CGT-GGG-TAG-AGT-CAT-ACT-GGA-ACA-T-3′	

### Quantitative Real-time PCR Analysis of TGF-β1, IL-1β, and Type IV Collagen Gene Expression

Quantitative real-time PCR (qPCR) was performed using SYBR Green qPCR Mix (Toyobo, Tokyo, Japan) and analyzed on a LightCycler 1.5 (Roche Diagnostics, Indianapolis, IN, USA). The genes selected were transforming growth factor (TGF)-β1 (*TGF-β1*), type IV collagen, and interleukin (IL)-1β (*IL-1β*) (Table 1). qPCR amplification was performed by incubation for 10 min at 95°C followed by 45 cycles of 10 s at 95°C, 10 s at 60°C, and 10 s at 72°C, and a final dissociation step at 65°C for 15 s. The crossing point of each sample was automatically determined by the LightCycler program, and the relative change ratio was determined using the ratio of the mRNA for the selected gene to that of glyceraldehyde-3-phosphate dehydrogenase [10]. PCR analysis was performed in duplicate.

### Fluorescence in situ Hybridization (FISH) Analysis of the Y Chromosome

To detect donor BM cells in recipient kidneys, the Y chromosome was detected by FISH in female rats. Cy3-labeled rat Y chromosomes (ID Labs Inc, London, Ontario, Canada) were provided in the supplier’s hybridization mix. FISH analysis was performed using IDetect™ according to the manufacturer’s recommendations. The Y-chromosome positive cells were calculated for ten randomly selected glomeruli of each kidney section. Images were obtained on an ECLIPSE 80i microscope equipped with an iAi progressive scan camera (Nikon, Tokyo, Japan) and Cytovision software (Applied Imaging, Newcastle, UK).

### Immunohistochemical Staining for ED-1 and G-CSFR

To confirm the presence of macrophages in glomeruli, immunohistochemical staining was performed on the kidneys of female rats that had received BM from male rats. We used a mouse monoclonal anti-monocyte/macrophage (ED-1) (1∶100 dilution; Serotec, Oxford, UK) or rabbit polyclonal anti-TGF-β1 (1∶200 dilution; Santa Cruz Biotechnology Inc., Santa Cruz, CA, USA) antibodies as the primary antibody. ED-1 and TGF-β1 levels were detected with streptavidin-peroxidase and peroxidase substrate solution (Dako, Copenhagen, Denmark). For ED-1, the numbers of positively stained macrophages were counted under high magnification (x400) in 120 to 130 glomeruli of each kidney section and an average score was calculated and expressed as positive cells/glomerulus. TGF-*β*1 levels were measured under high magnification x200 in ten images of each kidney section by image analysis using Image-Pro Plus 4.5 (Media Cybernetics, Silver Spring, MD) [28]. Images were obtained on a Leica DM4000B microscope equipped with a Leica DFC310 FX camera and LAS Basic V3.8 software (Leica Microsystems, Wetzlar, Germany).To identify G-CSFR in the glomeruli of each kidney section, we performed immunofluorescence staining. Tissue sections were incubated for 90 min with a mouse monoclonal anti-G-CSFR antibody as the primary antibody (1∶100 dilution; Santa Cruz Biotechnology Inc., Santa Cruz, CA, USA). The sections were washed and then incubated with fluorescein isothiocyanate-conjugated secondary antibody (1∶500 dilution; Abcam, Cambridge, MA, USA) for 60 min. Images were obtained on an ECLIPSE 80i microscope equipped with an iAi progressive scan camera (Nikon, Tokyo, Japan) and CytoVision system software (Applied Imaging, Newcastle, UK).

### Statistical Analysis

All data are presented as mean±SD, except for histological data, which are presented as mean±SE. Statistical differences were determined with the Statistical Package for the Social Sciences (SPSS) 18.0 software (SPSS Inc., Chicago, IL, USA). Data were analyzed using Mann Whitney U-tests (for single comparisons) or Kruskal-Wallis nonparametric ANOVA (for multiple comparisons). Values of *P*<0.05 were considered statistically significant.

## Results

### Development of the DN Model

**Figure 2:**
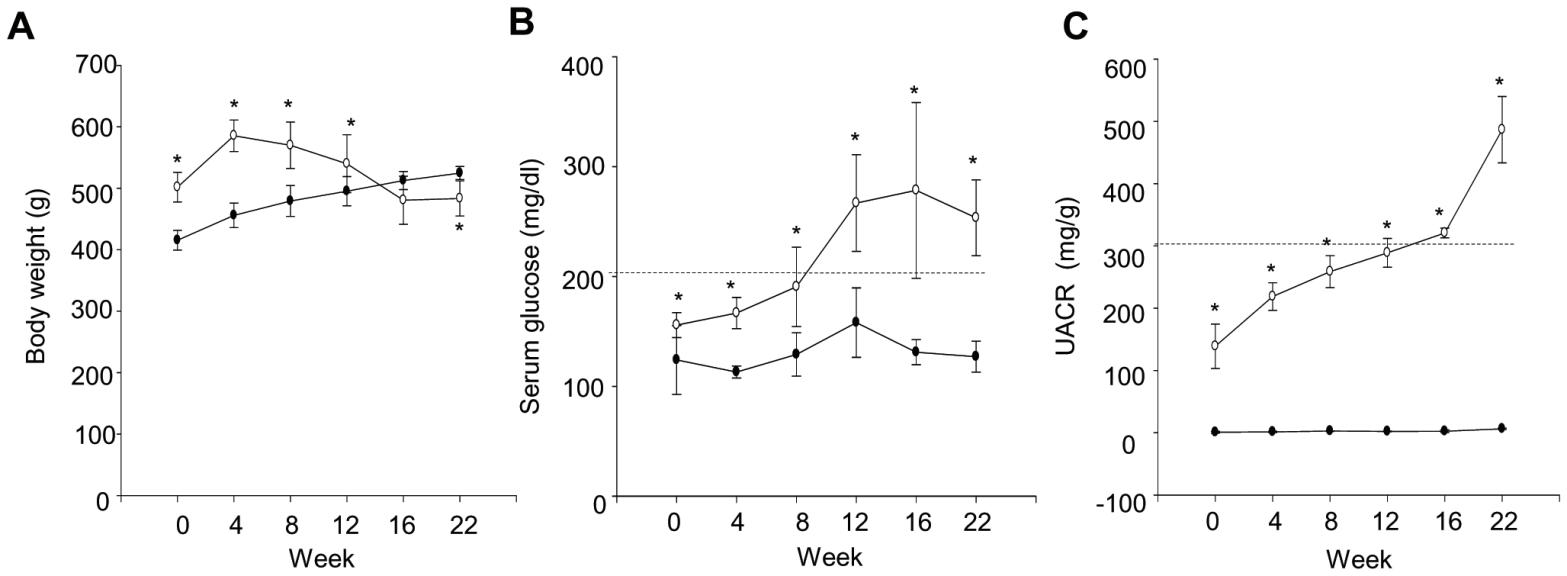
Changes of body weight, serum glucose, and UACR prior to treatment. Body weight (**A**), plasma glucose (**B**), and urine albumin creatinine ratio (UACR) (**C**) in rats. White circles: OLETF rats (n = 12) black circles: LETO rats (n = 7). All data are expressed as mean±SD. *P<0.05 vs. LETO rats.

**Figure 3:**
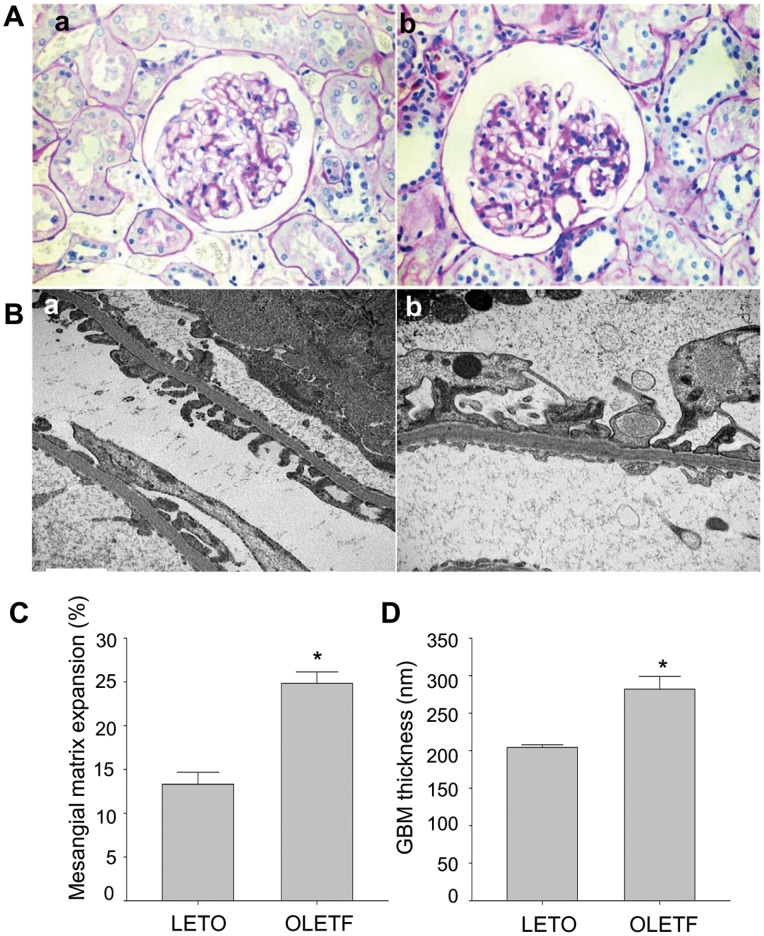
Histological changes in kidneys before treatment. (**A**) Stained with periodic acid-Schiff (PAS) (magnification x400). (**B**) Stained electron micrograph of a glomerulus (magnification x20.000). Kidney of the LETO rat (a) and the OLETF rat (b). (**C**) Quantitative analysis of PAS-stained kidney sections. (**D**) Quantitative analysis of GBM thickness via electron micrographs. All data are expressed as mean±SE. *P<0.05 vs. LETO rats (n = 3).

Body weight was significantly higher (*P*<0.05) in the OLETF rats than the control LETO rats during the first 12 weeks of sucrose feeding (to 28 weeks of age) but was significantly lower (*P*<0.05) after 22 weeks of sucrose feeding (at 38 weeks of age) (Fig. 2A). Serum glucose and UACR levels were higher in the OLETF rats than in the control LETO rats at all times (*P*<0.05). Serum glucose levels exceeded 200 mg/dl during the first 12 weeks of sucrose feeding and UACR levels exceeded 300 mg/g during the first 16 weeks of sucrose feeding (at 32 weeks of age) (Fig. 2B and C). Kidney/body weight, serum glucose, and UACR levels before treatment were higher in OLETF rats than the control LETO rats (*P*<0.05). CrCl levels did not differ between the two groups. Body weight was lower in OLETF rats than the control LETO rats (*P*<0.05). TC and TG levels were higher in OLETF rats than the control LETO rats (*P*<0.05) (Table 2). Histologically, mesangial matrix expansion and GBM thickness before treatment were higher in the OLETF rats than the control LETO rats (*P*<0.05) (Fig. 3). These changes were evidences of early DN.

**Table 2:** Levels of metabolic parameters before treatment with G-CSF or saline.

	LETO	OLETF
Body weight, g	530.75±9.84	475.13±30.64*
Kidney/body weight, %	0.29±0.00	0.39±0.01*
Serum glucose, mg/dl	118.50±4.04	264.75±23.92*
TC, mg/dl	94.50±6.76	176.38±40.33*
TG, mg/dl	38.00±9.83	142.63±31.10*
UACR, mg/g	6.41±0.34	486.59±30.79*
CrCl, ml/min	1.69±0.78	1.55±0.27

Long-Evans Tokushima Otsuka rats, LETO; Otsuka Long-Evans Tokushima Fatty rats, OLETF; total cholesterol, TC; triglyceride, TG; urine albumin creatinine ratio, UACR. All data are expressed as mean±SD. *P<0.05 vs. LETO rat (LETO, *n* = 4; OLETF, *n* = 8).

### Metabolic Parameters

Kidney/body weights and UACR after 4 weeks of treatment were significantly lower in the G-CSF-treated group than in the saline-treated group (*P*<0.05) but still higher than the control LETO group. TC levels were lower in the G-CSF-treated group than in the saline-treated group (*P*<0.05) and not significantly different from that of the control LETO group. Serum glucose, TG level, and body weight were not significantly different from in the saline-treated group. CrCl levels did not differ significantly within the three groups (Table 3).

**Table 3:** Levels of metabolic parameters after treatment with G-CSF or saline.

	LETO	OLETF+saline	OLETF+G-CSF
Body weight, g	583.25±16.07	518.36±36.65	547.62±30.41*
Kidney/body weight, %	0.24±0.04	0.44±0.03*	0.36±0.03*^,†^
Serum glucose, mg/dl	214.50±10.21	410.75±18.57*	427.00±12.19*
TC, mg/dl	104.25±5.25	265.00±22.02*	161.75±55.69^†^
TG, mg/dl	49.50±4.20	185.00±62.89*	123.50±23.27*
UACR, mg/g	8.42±7.58	648.77±72.33*	451.00±122.84*^,†^
CrCl, ml/min	1.64±0.18	1.81±0.37	1.68±0.47

Long-Evans Tokushima Otsuka rats, LETO; Otsuka Long-Evans Tokushima Fatty rats, OLETF; total cholesterol, TC; triglyceride, TG; urine albumin creatinine ratio, UACR. All data are expressed as mean±SD. *P<0.05 vs. LETO rat. ^†^ *P*<0.05 vs. untreated OLETF rat (*n* = 4).

### Histological Findings

**Figure 4:**
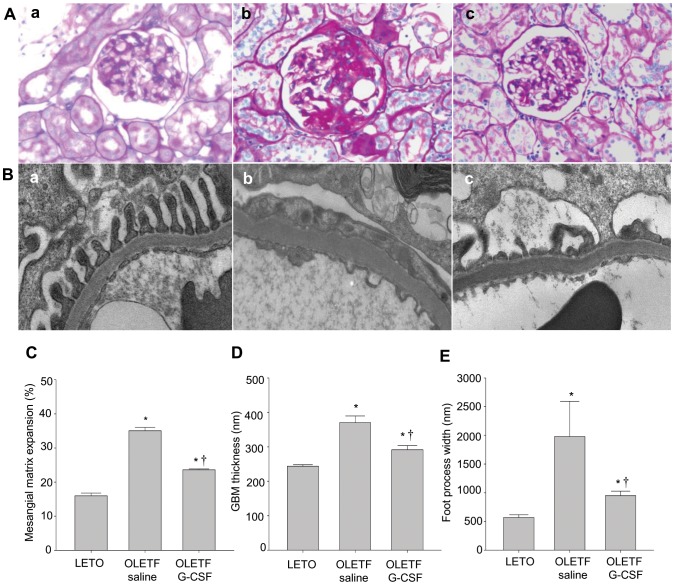
Histological changes in the kidney after treatment. (**A**) Stained with periodic acid-Shiff (PAS) (magnification x400). (**B**) Electron micrograph of a glomerulus (magnification x20.000). Kidney of the LETO rat (a), the saline-treated OLETF rat (b), and the G-CSF-treated OLETF rat (c). (**C**) Quantitative analysis of images of PAS-stained kidney sections. (**D**) Quantitative analysis of images of GBM thickness via electron micrographs. (**E**) Quantitative analysis of foot process width via electron micrographs. All data are expressed as mean±SE. *P<0.05 vs. LETO rats. ^†^ *P*<0.05 vs. untreated OLETF rats (n = 4).

Mesangial matrix expansion after 4 weeks of treatment was lower in the G-CSF-treated group than in the saline-treated group (*P*<0.05) but still higher than the control LETO group (*P*<0.05) (Fig. 4A and C). In addition, electron microscopic examination revealed irregular GBM thickening and podocyte foot process effacements in some parts of the glomeruli (Fig. 4B). GBM thickness and FPW were lower in the G-CSF-treated group than in the saline-treated group (*P*<0.05) and not significantly different from that of the control LETO group (Fig. 4D). FPW was lower in the G-CSF-treated group than in the saline-treated group (*P*<0.05) but still higher than the control LETO group (*P*<0.05) (Fig. 4E).

### Expression Levels of TGF-β1, Type IV Collagen, and IL-1β after Treatment

**Figure 5:**
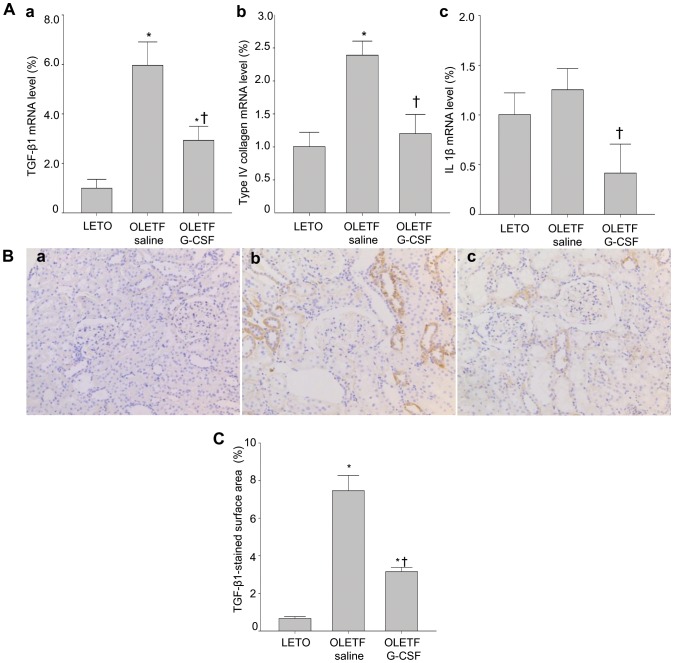
Expression levels of TGF-β1, type IV collagen, and IL-1β after treatment. (**A**) Levels of TGF-β1 (a), type IV collagen (b), and IL-1β (c) mRNA expression were determined using quantitative real-time PCR. (**B**) Level of TGF-β1 protein expression was determined using immunohistochemical staining. (**C**) Quantitative analysis of TGF-β1 protein expression. Mean values were calculated from the kidneys of three separate animals. Changes were determined relative to glyceraldehyde-3-phosphate dehydrogenase. All data are expressed as mean±SD. *P<0.05 vs. LETO rats. ^†^ *P*<0.05 vs. untreated OLETF rats (n = 3).

The level of TGF-β1 mRNA was lower in the G-CSF-treated group than in the saline-treated group (*P*<0.05) but still higher than the control LETO group (*P*<0.05) Levels of type IV collagen and IL-1β mRNA were also lower in the G-CSF-treated group than in the saline-treated group (*P*<0.05) and not significantly different from those of the control LETO group (Fig. 5A). Immunohistochemical staining for TGF-β1 and subsequent quantitative analysis demonstrated that TGF-β1 protein expression was lower in the G-CSF-treated group than in the saline-treated group (*P*<0.05) (Fig. 5B and C).

### Mechanism of the G-CSF Effect - Y Chromosome-positive Cells within Glomeruli

**Figure 6:**
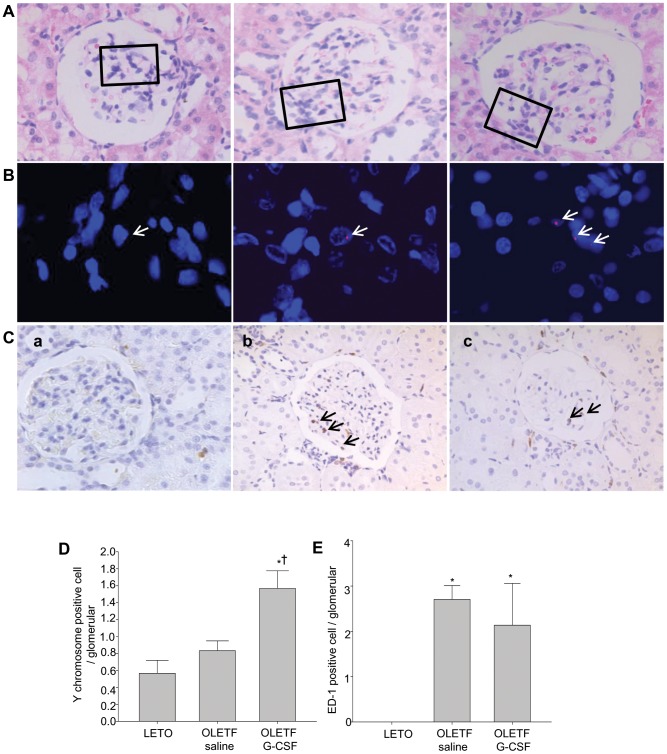
FISH imaging and immunostained ED-1 in glomeruli of BMT female rats after treatment. (**A**) Stained with hematoxylin and eosin (HE) (magnification x400). (**B**) Higher magnification views of the boxed regions in (A), stained with FISH using a Cy3-labeled Y-chromosome (red, white arrow) and DAPI-labeled nucleus (blue) (magnification x400). (**C**) Macrophages immunostained with ED-1 antibody (black arrow). Kidney of the LETO rat (a), the saline-treated OLETF rat (b), and the G-CSF-treated OLETF rat (c). (**D**) Quantitative analysis of Y-chromosome-positive cells in glomeruli. (**E**) Quantitative analysis of ED-1-positive cells in glomeruli. Fluorescence *in situ* hybridization, FISH; 4′–6-Diamidino-2-phenylindole, DAPI; Bone marrow transplantation, BMT. All data are expressed as mean±SD. *P<0.05 vs. LETO rats. ^†^ *P*<0.05 vs. untreated OLETF rats (n = 3).

Y chromosome-positive cells were detected in the glomeruli of the kidney tissue of female rats (Fig. 6A and B). The numbers of Y chromosome cells were higher in the G-CSF-treated group than in the saline-treated group (*P*<0.05) (Fig. 6D). In addition, immunohistochemical staining for ED-1 showed that there were no significant differences in macrophage density in glomeruli between the G-CSF- and saline-treated groups (Fig. 6C and E).

### Mechanism of the G-CSF Effect - G-CSFR Analysis

**Figure 7:**
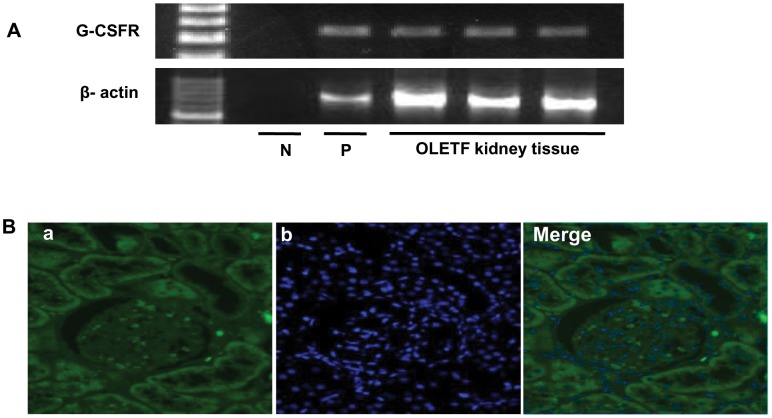
Expression of the G-CSF receptor (G-CSFR) in kidneys. (**A**) RT-PCR analysis of G-CSFR mRNA expression in kidney tissue. Hypothalamus tissue was used as a positive control, together with a no-template negative control. (**B**) G-CSFR immunostained via antibody (green, a) and DAPI (blue, b) in glomeruli of a kidney section (magnification x400). G-CSF receptor, G-CSFR; positive control, P; negative control, N.

We confirmed the presence of G-CSFR in kidney tissue by RT-PCR (Fig. 7A). However, immunofluorescence analysis revealed that it was not present in the glomeruli (Fig. 7B).

## Discussion

Our data show that G-CSF treatment can prevent progression of early DN in OLETF rats, and that mobilization of BM cells, rather than a direct effect of G-CSF, is the likely explanation for the observed effect.

Previous studies have demonstrated that the characteristic glomerular changes in DN include mesangial matrix expansion, GBM thickening, and podocyte foot process effacement [29]. In our study, the beneficial effects of G-CSF in DN were evident from the histological findings. We confirmed by PAS staining that G-CSF reduced mesangial matrix expansion in kidney tissue. We also confirmed by electron micrographs that G-CSF decreased GBM thickness and podocyte foot process effacement in some parts of the glomeruli. Functionally, we showed that G-CSF decreased UACR level.

According to a previous study, early DN is characterized by increased kidney/body weight and UACR levels and unchanged CrCl level [30]. In our model, kidney/body weight and UACR levels increased, whereas the CrCl level did not change, thus showing that the model could be characterized as early DN.

The pathophysiology of DN involves the accumulation of microvascular matrix protein and inflammation. Previous studies indicated that TGF-β1 played a key role in the development of renal hypertrophy and the accumulation of extracellular matrix components in diabetes, as well as stimulation of the synthesis of extracellular matrix molecules such as type IV collagen [31], [32]. Further, inflammatory cytokines such as IL-1β caused inflammation by increasing the expression and synthesis of adhesion molecules [33]. We demonstrated that G-CSF clearly reduced TGF-β1, type IV collagen, and IL-1β mRNA expression.

Previous studies suggested several possible mechanisms for the general effect of G-CSF on renal disease. First, G-CSF may affect the kidney directly. A direct effect of G-CSF on cardiomyocytes and endothelial cells has been reported [34], [35]. In the kidney, only a direct effect of G-CSF on renal tubules, in which express G-CSFR, has been reported [36]. We found by RT-PCR that G-CSFR was present in kidney tissue; however, immunofluorescence analysis revealed that it was not present in mesangial cells, podocytes, or endothelial cells in glomeruli. In this study, we observed that the therapeutic effects of G-CSF, including a decrease in GBM thickness and podocyte foot process effacement, appeared in glomeruli. There results suggest that G-CSF does not directly act through G-CSFR in glomerular cells because G-CSFR is not expressed at mesangial cells, podocytes, and endothelial cells in glomeruli.

As another possible mechanism, increased bone marrow cells homing to injured renal cells could induce trans-differentiation or trophic effects, thereby contributing to renal cell repair. Previous reports suggested that BM cells might trans-differentiate into renal tubular cells that repair kidneys [37]. Prodromidi et al. demonstrated that podocyte regeneration in a renal disease model was improved by paracrine action of bone marrow-derived cells [38]. In addition, the mechanism of G-CSF-induced hematopoietic stem cell mobilization by stromal cell-derived factor (SDF)-1 and CXCP4 interaction was well known [39]. In our sex-mismatched BMT study, Y chromosome-positive cells were found in glomeruli. The number of these cells was higher in the G-CSF-treated group than that in the saline-treated group. These findings indicate that BM cells are mobilized to damaged kidney tissue by G-CSF. Early reports showed that BM cells decreased inflammation in rats with acute myocardial infarction [40], and BM-derived mesenchymal stem cells suppressed inflammation through secretion of anti-inflammatory cytokines [41]. In addition, macrophage numbers did not differ significantly between the G-CSF- and saline-treated groups. These findings indicate that BM-derived cells are mobilized to damaged kidney tissue by G-CSF, as well as other cells such as macrophages, stem cells, and blood cells. Based on our results, we suggest that G-CSF prevents the progression of DN by mobilizing BM cells, rather than by a direct effect, because G-CSFR is absent from glomeruli and BM cells are mobilized to damaged kidney tissue by G-CSF.

Sugimoto et al. have demonstrated that administration of pioglitazone for 6 months ameliorates renal injury, and Ko et al. showed that treatment with enalapril for 32 weeks had beneficial effects on renal damage due to diabetes [15], [42]. We have confirmed that a relatively short G-CSF treatment (5 days) is effective and does not have severe side effects [43]. For this reason, G-CSF may be a promising drug for the treatment of DN.

This study has three limitations. First, investigating the main mechanism underlying the effect of G-CSF on early DN will need further investigations. To clarify the mechanism, illustration of a BM-dependent effect of G-CSF and analysis about downstream G-CSFR targets such as hematopoietic cell-specific Lyn substrate (HCLS)1, HCLS1-associated protein X (HAX)1, and lymphoid-enhancer binding factor (Lef)-1 in order to confirm no direct effect of G-CSF on early DN are needed. Second, the effects of G-CSF on the different stages of DN were not demonstrated. Finally, we did not establish the optimum dosage and regimen of G-CSF. In order to overcome this limitation, future studies should involve a larger number of animals.

In summary, we demonstrate that G-CSF could prevent the progression of early DN in OLETF rats. In addition, we speculate that mobilization of BM cells, rather than a direct effect of G-CSF, could be the mechanism of the observed G-CSF effect. This is the first report to show beneficial effects of G-CSF on early DN in an animal model. Our findings suggest that G-CSF has potential as a novel therapeutic drug in early DN patients.

## References

[1] Alsaad KO, Herzenberg AM (2007) Distinguishing diabetic nephropathy from other causes of glomerulosclerosis: an update. J Clin Pathol 60: 18–26. 17213346 10.1136/jcp.2005.035592 PMC1860608

[2] Giunti S, Barit D, Cooper ME (2006) Mechanisms of diabetic nephropathy: role of hypertension. Hypertension 48: 519–526. 16952978 10.1161/01.HYP.0000240331.32352.0c

[3] Fukuzawa Y, Watanabe Y, Inaguma D, Hotta N (1996) Evaluation of glomerular lesion and abnormal urinary findings in OLETF rats resulting from a long-term diabetic state. J Lab Clin Med 128: 568–578. 8960640 10.1016/s0022-2143(96)90129-8

[4] Adler S (1994) Structure-function relationships associated with extracellular matrix alterations in diabetic glomerulopathy. J Am Soc Nephrol 5: 1165–1172. 7873725 10.1681/ASN.V551165

[5] Mauer SM, Steffes MW, Ellis EN, Sutherland DE, Brown DM, et al (1984) Structural-functional relationships in diabetic nephropathy. J Clin Invest 74: 1143–1155. 6480821 10.1172/JCI111523 PMC425280

[6] Zohlnhofer D, Ott I, Mehilli J, Schomig K, Michalk F, et al (2006) Stem cell mobilization by granulocyte colony-stimulating factor in patients with acute myocardial infarction: a randomized controlled trial. JAMA 295: 1003–1010. 16507801 10.1001/jama.295.9.1003

[7] Sheridan WP, Morstyn G, Wolf M, Dodds A, Lusk J, et al (1989) Granulocyte colony-stimulating factor and neutrophil recovery after high-dose chemotherapy and autologous bone marrow transplantation. Lancet 2: 891–895. 2477656 10.1016/s0140-6736(89)91552-3

[8] Lee ST, Chu K, Jung KH, Ko SY, Kim EH, et al (2005) Granulocyte colony-stimulating factor enhances angiogenesis after focal cerebral ischemia. Brain Res 1058: 120–128. 16150422 10.1016/j.brainres.2005.07.076

[9] Hou XW, Jiang Y, Wang LF, Xu HY, Lin HM, et al (2009) Protective role of granulocyte colony-stimulating factor against adriamycin induced cardiac, renal and hepatic toxicities. Toxicol Lett 187: 40–44. 19429242 10.1016/j.toxlet.2009.01.025

[10] Yi-Sun S, Cheng-Hu F, Byung-Im S, Jun-Young P, Dae Won J, et al (2013) Therapeutic effects of granulocyte-colony stimulating factor on non-alcoholic hepatic steatosis in the rat. Ann Hepatol 12: 115–122. 23293202

[11] Lim YH, Joe JH, Jang KS, Song YS, So BI, et al (2011) Effects of granulocyte-colony stimulating factor (G-CSF) on diabetic cardiomyopathy in Otsuka Long-Evans Tokushima fatty rats. Cardiovasc Diabetol 10: 92. 21999467 10.1186/1475-2840-10-92 PMC3215959

[12] Flaquer M, Franquesa M, Vidal A, Bolanos N, Torras J, et al (2012) Hepatocyte growth factor gene therapy enhances infiltration of macrophages and may induce kidney repair in db/db mice as a model of diabetes. Diabetologia 55: 2059–2068. 22460762 10.1007/s00125-012-2535-z PMC3369134

[13] Kilkenny C, Browne WJ, Cuthi I, Emerson M, Altman DG (2012) Improving bioscience research reporting: the ARRIVE guidelines for reporting animal research. Vet Clin Pathol 41: 27–31. 22390425 10.1111/j.1939-165X.2012.00418.x

[14] Shoji E, Okumura T, Onodera S, Takahashi N, Harada K, et al (1997) Gastric emptying in OLETF rats not expressing CCK-A receptor gene. Dig Dis Sci 42: 915–919. 9149042 10.1023/a:1018860313674

[15] Sugimoto K, Tsuruoka S, Fujimura A (2001) Effect of enalapril on diabetic nephropathy in OLETF rats: the role of an anti-oxidative action in its protective properties. Clin Exp Pharmacol Physiol 28: 826–830. 11553023 10.1046/j.1440-1681.2001.03530.x

[16] Bennett PH, Haffner S, Kasiske BL, Keane WF, Mogensen CE, et al (1995) Screening and management of microalbuminuria in patients with diabetes mellitus: recommendations to the Scientific Advisory Board of the National Kidney Foundation from an ad hoc committee of the Council on Diabetes Mellitus of the National Kidney Foundation. Am J Kidney Dis 25: 107–112. 7810516 10.1016/0272-6386(95)90636-3

[17] Ziyadeh FN (1993) The extracellular matrix in diabetic nephropathy. Am J Kidney Dis 22: 736–744. 8238022 10.1016/s0272-6386(12)80440-9

[18] Alpdogan O, Schmaltz C, Muriglan SJ, Kappel BJ, Perales MA, et al (2001) Administration of interleukin-7 after allogeneic bone marrow transplantation improves immune reconstitution without aggravating graft-versus-host disease. Blood 98: 2256–2265. 11568014 10.1182/blood.v98.7.2256

[19] Ho CC, Hau PM, Marxer M, Poon RY (2010) The requirement of p53 for maintaining chromosomal stability during tetraploidization. Oncotarget 1: 583–595. 21317454 10.18632/oncotarget.193 PMC3248137

[20] Tai CY, Strande LF, Eydelman R, Sheng X, VanTran JL, et al (2004) Absence of graft-versus-host disease in the isolated vascularized bone marrow transplant. Transplantation 77: 316–319. 14743002 10.1097/01.TP.0000101511.11171.EF

[21] Song YS, Fang CH, So BI, Park JY, Jun DW, et al (2013) Therapeutic effects of granulocyte-colony stimulating factor on non-alcoholic hepatic steatosis in the rat. Ann Hepatol 12: 115–122. 23293202

[22] Kim SY, Lim AY, Jeon SK, Lee IS, Choue R (2011) Effects of dietary protein and fat contents on renal function and inflammatory cytokines in rats with adriamycin-induced nephrotic syndrome. Mediators Inflamm 2011: 945123. 21822358 10.1155/2011/945123 PMC3136151

[23] Blom D, Yamin TT, Champy MF, Selloum M, Bedu E, et al (2010) Altered lipoprotein metabolism in P2Y(13) knockout mice. Biochim Biophys Acta 1801: 1349–1360. 20817122 10.1016/j.bbalip.2010.08.013

[24] Zhang HM, Dang H, Kamat A, Yeh CK, Zhang BX (2012) Geldanamycin derivative ameliorates high fat diet-induced renal failure in diabetes. PLoS One 7: e32746. 22412919 10.1371/journal.pone.0032746 PMC3295767

[25] Thomson SE, McLennan SV, Kirwan PD, Heffernan SJ, Hennessy A, et al (2008) Renal connective tissue growth factor correlates with glomerular basement membrane thickness and prospective albuminuria in a non-human primate model of diabetes: possible predictive marker for incipient diabetic nephropathy. J Diabetes Complications 22: 284–294. 18413184 10.1016/j.jdiacomp.2007.07.001

[26] Deegens JK, Dijkman HB, Borm GF, Steenbergen EJ, van den Berg JG, et al (2008) Podocyte foot process effacement as a diagnostic tool in focal segmental glomerulosclerosis. Kidney Int 74: 1568–1576. 18813290 10.1038/ki.2008.413

[27] van den Berg JG, van den Bergh Weerman MA, Assmann KJ, Weening JJ, Florquin S (2004) Podocyte foot process effacement is not correlated with the level of proteinuria in human glomerulopathies. Kidney Int 66: 1901–1906. 15496161 10.1111/j.1523-1755.2004.00964.x

[28] Belmiro CL, Goncalves RG, Kozlowski EO, Werneck AF, Takyia CM, et al (2011) Dermatan sulfate reduces monocyte chemoattractant protein 1 and TGF-beta production, as well as macrophage recruitment and myofibroblast accumulation in mice with unilateral ureteral obstruction. Braz J Med Biol Res 44: 624–633. 21833458 10.1590/s0100-879x2011007500077

[29] Jefferson JA, Shankland SJ, Pichler RH (2008) Proteinuria in diabetic kidney disease: a mechanistic viewpoint. Kidney Int 74: 22–36. 18418356 10.1038/ki.2008.128

[30] Mogensen CE (1987) Microalbuminuria as a predictor of clinical diabetic nephropathy. Kidney Int 31: 673–689. 3550239 10.1038/ki.1987.50

[31] Wolf G, Ziyadeh FN (1999) Molecular mechanisms of diabetic renal hypertrophy. Kidney Int 56: 393–405. 10432377 10.1046/j.1523-1755.1999.00590.x

[32] Ziyadeh FN (2004) Mediators of diabetic renal disease: the case for tgf-Beta as the major mediator. J Am Soc Nephrol 15 Suppl 1S55–57. 14684674 10.1097/01.asn.0000093460.24823.5b

[33] Navarro-Gonzalez JF, Mora-Fernandez C (2008) The role of inflammatory cytokines in diabetic nephropathy. J Am Soc Nephrol 19: 433–442. 18256353 10.1681/ASN.2007091048

[34] Harada M, Qin Y, Takano H, Minamino T, Zou Y, et al (2005) G-CSF prevents cardiac remodeling after myocardial infarction by activating the Jak-Stat pathway in cardiomyocytes. Nat Med 11: 305–311. 15723072 10.1038/nm1199

[35] Park KW, Kwon YW, Cho HJ, Shin JI, Kim YJ, et al (2008) G-CSF exerts dual effects on endothelial cells–opposing actions of direct eNOS induction versus indirect CRP elevation. J Mol Cell Cardiol 45: 670–678. 18675273 10.1016/j.yjmcc.2008.07.002

[36] Wei Q, Hill WD, Su Y, Huang S, Dong Z (2011) Heme oxygenase-1 induction contributes to renoprotection by G-CSF during rhabdomyolysis-associated acute kidney injury. Am J Physiol Renal Physiol 301: F162–170. 21511696 10.1152/ajprenal.00438.2010 PMC3129892

[37] Fang TC, Alison MR, Cook HT, Jeffery R, Wright NA, et al (2005) Proliferation of bone marrow-derived cells contributes to regeneration after folic acid-induced acute tubular injury. J Am Soc Nephrol 16: 1723–1732. 15814835 10.1681/ASN.2004121089

[38] Prodromidi EI, Poulsom R, Jeffery R, Roufosse CA, Pollard PJ, et al (2006) Bone marrow-derived cells contribute to podocyte regeneration and amelioration of renal disease in a mouse model of Alport syndrome. Stem Cells 24: 2448–2455. 16873763 10.1634/stemcells.2006-0201

[39] Petit I, Szyper-Kravitz M, Nagler A, Lahav M, Peled A, et al (2002) G-CSF induces stem cell mobilization by decreasing bone marrow SDF-1 and up-regulating CXCR4. Nat Immunol 3: 687–694. 12068293 10.1038/ni813

[40] Tavares AM, da Rosa Araujo AS, Baldo G, Matte U, Khaper N, et al (2010) Bone marrow derived cells decrease inflammation but not oxidative stress in an experimental model of acute myocardial infarction. Life Sci 87: 699–706. 20970437 10.1016/j.lfs.2010.10.008

[41] Ryan JM, Barry FP, Murphy JM, Mahon BP (2005) Mesenchymal stem cells avoid allogeneic rejection. J Inflamm (Lond) 2: 8. 16045800 10.1186/1476-9255-2-8 PMC1215510

[42] Ko GJ, Kang YS, Han SY, Lee MH, Song HK, et al (2008) Pioglitazone attenuates diabetic nephropathy through an anti-inflammatory mechanism in type 2 diabetic rats. Nephrol Dial Transplant 23: 2750–2760. 18388116 10.1093/ndt/gfn157

[43] Gabrilove JL, Jakubowski A, Scher H, Sternberg C, Wong G, et al (1988) Effect of granulocyte colony-stimulating factor on neutropenia and associated morbidity due to chemotherapy for transitional-cell carcinoma of the urothelium. N Engl J Med 318: 1414–1422. 2452983 10.1056/NEJM198806023182202

